# Legalizing Dissection

**Published:** 1874-03

**Authors:** 


### LEGALIZING DISSECTION.

AFTER repeated asking, and long waiting, those engaged in medical teaching in this State have finally secured the passage of a law authorizing dissections, and providing a legal mode of obtaining the necessary material. We tender the thanks of the profession to Drs. Wilcox, Mitchell, Rice, Collins, Rogers, and other members of the Legislature, who efficiently and perseveringly worked for the passage of the bill. The following is a correct copy of the law as it passed both branches of the Legislature and received the signature of the Governor:

*A Bill for an Act to Promote the Science of Medicine and Surgery in the State of Illinois:*

SECTION I. *Be it Enacted by the People of the State of Illinois, Represented in the General Assembly,* It shall be lawful, in cities and counties whose population exceeds one hundred thousand inhabitants, for superintendents of penitentiaries, wardens of poorhouses, coroners and city undertakers, to deliver to the professors and teachers in medical colleges and schools in this State, and for professors and teachers to receive, the remains or body of any deceased person, for purposes of medical and surgical study: *Provided,* that said remains shall not have been regularly interred, and shall not have been desired for interment by any relatives or friends of said deceased within forty-eight hours after death: *Provided, also,* that the remains of no person, who may be known to have relatives or friends, shall be so delivered or received without the written consent of said relatives or friends: *And, provided, further,* that the remains of no one detained for debt, or as a witness, or on suspicion of crime, or of any traveler, or of any person who shall have expressed a desire, in his or her last sickness, that his or her body may be interred, shall be delivered or received as aforesaid, but shall be buried in the usual manner: *And, provided, also,* that in case the remains of any person so delivered or received shall be subsequently claimed by any surviving relative or friend for interment: *Provided, further,* that notice shall be given to friends or relatives of any deceased person, if such friends or relatives are known to the authorities.

§ 2. And it shall be the duty of the said professors and teachers decently to bury, in some public cemetery, the remains of all bodies, after they shall have answered the purposes of study aforesaid; and for any neglect or violation of the provisions of this act, the party so neglecting shall forfeit and pay a penalty of not less than fifty nor more than one hundred dollars, or be imprisoned in the county jail not less than six or more than twelve months, or both, at the discretion of the court; such penalties to be sued for by the health or school officers, or any person interested therein, for the benefit of the school fund or health department, as the case may be.

§ 3. The remains or bodies of said persons as may be so received by the professors and teachers, as aforesaid, shall be used for the purposes of medical and surgical study alone, and in this State only; and whoever shall use such remains for any other purpose, or shall remove such remains beyond the limits of this State, or in any manner traffic in the same, or in any manner aid or assist in the same, shall be deemed guilty of a misdemeanor, and shall, on conviction, be imprisoned for a term not exceeding one year in a county jail.

§ 4. Every person who shall deliver up the remains of any deceased person, in violation of or contrary to any or all of the provisions contained in the first section of this act, and every person who shall receive said remains, knowing them to have been delivered contrary to any of the provisions of said section, shall, each and every one of them, be deemed guilty of a misdemeanor, and shall, on conviction, be imprisoned for a term not exceeding two years in a county jail.

